# Supplementary figures and images for: Accelerated DNA methylation aging and increased resilience in veterans: The biological cost for soldiering on
Source: Neurobiol Stress. 2018 Apr 7;8:112–9. doi: 10.1016/j.ynstr.2018.04.001 (PMC5991315; doi:10.1016/j.ynstr.2018.04.001)

Non Graphical Solutions to Scree Test

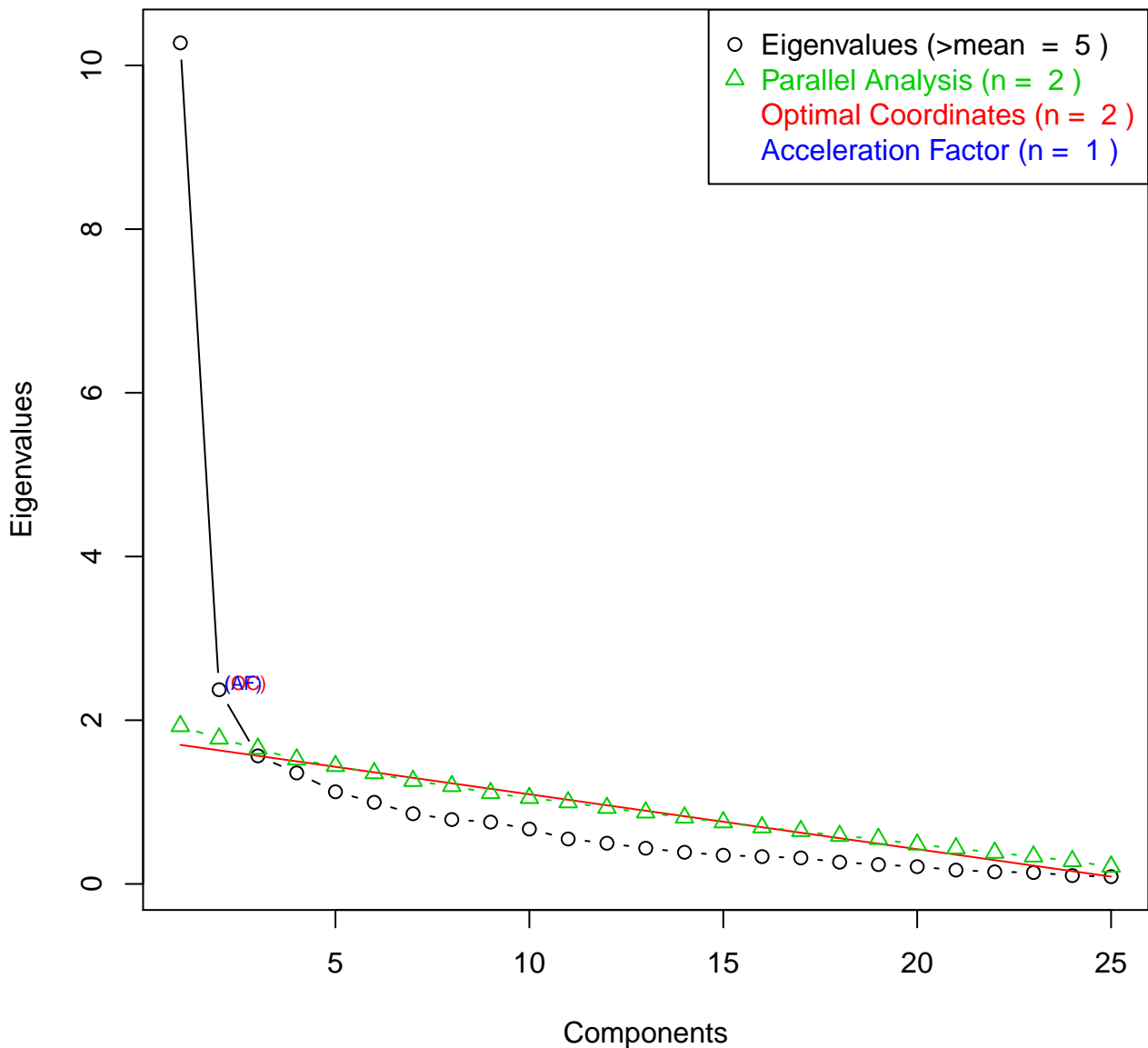

Supplement: Supplementary Fig. 1a NEW [file mmc1.pdf]

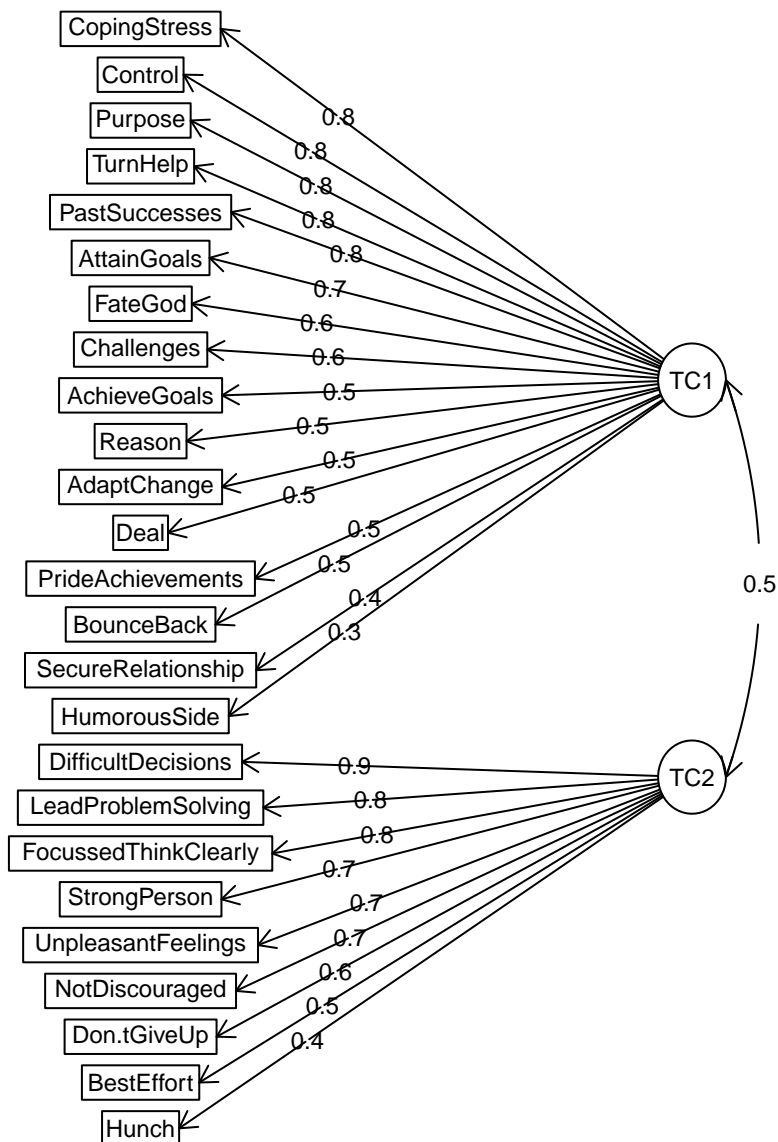

Supplement: Supplementary Fig. 1b NEW [file mmc2.pdf]
